# AHR-dependent changes in the mitochondrial proteome in response to 2,3,7,8-tetrachlorodibenzo-p-dioxin

**DOI:** 10.1016/j.dib.2016.05.023

**Published:** 2016-05-20

**Authors:** Hye Jin Hwang, Peter Dornbos, John J. LaPres

**Affiliations:** a Department of Biochemistry and Molecular Biology, Michigan State University, East Lansing, MI 48824, United States; b Center for Mitochondrial Science and Medicine, Michigan State University, East Lansing, MI 48824, United States; c Institute for Integrative Toxicology, Michigan State University, East Lansing, MI 48824-1319, United States

**Keywords:** Proteomics, SILAC, TCDD, AHR, aryl hydrocarbon receptor, Mitochondria

## Abstract

The aryl hydrocarbon receptor (AHR) is a ligand-activated transcription factor that is the principal regulator of a cell׳s response to many polyaromatic hydrocarbons, such as 2,3,7,8-tetrachlorodibenzo-p-dioxin (TCDD). To gain a better understanding of the impact of TCDD on the mitochondrial proteome, a stable isotope labeling by amino acids in cell culture (SILAC)-based proteomic analysis was performed. We used two mouse hepatoma cell lines that differ in AHR expression levels, hepa1c1c7 (AHR-expressing) and hepac12 (AHR-deficient). The cell lines were exposed to TCDD (10 nM) for 72 h; each treatment was assayed in triplicate and were analyzed as separate runs on the mass-spectrometer. Mitochondria were then isolated and mitochondrial proteins were separated by SDS-PAGE and subject to mass spectrometry. The data presented were collected from four independent SILAC experiments. Within each experiment, three isotopes were employed to compare protein ratios via mass-spectrometry: (1) light l-arginine/l-lysine HCl (Arg0, Lys0), (2) medium ^15^N_4_-l-arginin/^13^C_6_l-lysine HCl (Arg4, Lys6), and (3) heavy ^13^C_6_^15^N_4_l-arginine/^13^C_6_^15^N_2_l-lysine HCl (Arg10, Lys8). The raw data includes approximately 2500 annotated proteins. The datasets provided by this study can be a reference to other toxicologists investigating TCDD-induced mitochondrial dysfunction. The data presented here are associated with the research article, “Mitochondrial-targeted Aryl Hydrocarbon Receptor and the Impact of 2,3,7,8-Tetrachlorodibenzo-p-Dioxin on Cellular Respiration and the Mitochondrial Proteome” (Hwang et al. (2016) [1]).

**Specifications Table**

**Table:** 

Subject area	Biochemistry, Toxicology
More specific subject area	Proteomics
Type of data	Figure, text files
How data was acquired	Thermo EASYnLC 1000 liquid chromatograph (Thermo Scientific) and ThermoFisher Q-Exactive mass spectrometer (Thermo Scientific)
Data format	Raw, MaxQuant txt format
Experimental factors	Proteomic analysis: hepa1c1c7 and hepac12 cells were cultured in light (Arg0, Lys0), medium (Arg4, Lys6), or heavy (Arg10, Lys8) media for 5 cell doublings, and subsequently treated with 10 nM TCDD or 0.01% DMSO for additional 72 h.
Experimental features	Mitochondria were isolated from each treatment and combined with equal amount of proteins (35 μg) labeled with light, medium, or heavy amino acids within one experimental set. When samples from the four independent experimental were collected, proteins were separated by SDS-PAGE and in-gel digested with trypin, and resulting peptides were analyzed by LC–MS/MS.
Data source location	East Lansing, MI, USA
Data accessibility	Data are within the article

**Value of the data**

• This is the first SILAC-based study that assesses AHR-dependent TCDD-induced changes in the mitochondrial proteome.
• This raw data, which identified 2500 annotated proteins, is a valuable resource for those interested in AHR-dependent changes in the mitochondrial proteome and, thereafter, mitochondrial function.
• As TCDD is known to induce metabolic syndrome, datasets such as these might provide mechanistic links between the pathology and AHR activation.
• The data was generated with a unique experimental design that, using 4 replicates with limited isotope labeling conditions, provides quantifiable changes in proteins in an untargeted manner.

## 1 Data

**Fig. 1:**
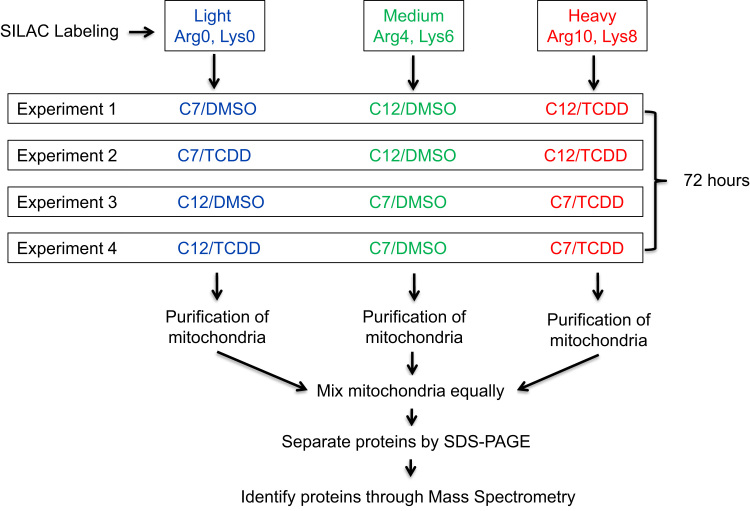
Workflow for SILAC-based proteomic analysis of TCDD-induced changes to the mitochondrial proteome in two mouse hepatoma cell lines, hepa1c1c7 (C7) and hepac12 (C12).

This article provides the data from a SILAC-based experiment aimed at characterizing the role of AHR in TCDD-induced changes in the mitochondrial proteome. Here, two mouse hepatoma cells having different levels of AHR expression, hepa1c1c7 (C7, AHR-expressing) and hepac12 (C12, AHR-deficient) were exposed to 10 nM TCDD or 0.01% DMSO for 72 h. To generate replicates of each treatment for downstream statistical analysis, four independent experiments using 3 distinguishing isotope labels were performed as shown in Fig. 1. This article includes four data files; each file contains the results for an independent experiment. Each data file is an output produced with MaxQuant software following identification and annotation of 2500 unique proteins. Furthermore, the MaxQuant software compares the quantity of the isotope labels for each identified protein in each independent experiment; such ratios indicate potential changes in expression that were induced by treatments (aka + or − TCDD) in each cell type (C7 and C12).

## 2 Experimental design, materials and methods

The experimental workflow is provided in Fig. 1.

### 2.1 Cell culture

Cell culture was performed as previously described [1].

### 2.2 SILAC

The SILAC experiment was performed as previously described [1]. Briefly, mouse hepatoma cells were grown in SILAC™-DMEM supplemented with 10% dialyzed fetal bovine serum, 1 mM sodium pyruvate, 2 mM l-glutamine, and distinct isotope-labeled amino acids supplements: 100 μg/mL l-arginine, and 100 *μ*g/mL l-lysine (light l-arginine/l-lysine HCl (Arg0, Lys0), medium ^15^N_4_-l-arginine/^13^C_6_l-lysine HCl (Arg4, Lys6), or heavy ^13^C_6_^15^N_4_l-arginine/^13^C_6_^15^N_2_l-lysine HCl (Arg10, Lys8) (SILAC™ media kit, Invitrogen and Cambridge Isotope Laboratories)) for 5 cell doublings to insure full incorporation of labeled amino acids into cellular proteins. Cells were then treated with 10 nM TCDD or 0.01% DMSO (vehicle control) for 72 h.

### 2.3 Preparation of mitochondrial proteins

TCDD or DMSO-treated cells were harvested and mitochondrial fractions were isolated using protocols adapted from previous reports [1], [2], [3].

### 2.4 Mass spectrometry (MS) and data analysis

Mass spectrometry was performed as previously described [1]. Briefly, SDS-PAGE gels were divided into 10 equal slices, and each slice was digested with trypsin as previously described with modifications [1], [4]. Peptides were extracted from the gel and dissolved in 2% acetonitrile/0.1% trifluoroacetic acid and automatically injected by a Thermo EASYnLC 1000 onto a Thermo Acclaim PepMap RSLC 0.075 mm×250 mm C18 column (Buffer A=99.9% Water/0.1% Formic Acid, Buffer B=99.9% Acetonitrile/0.1% Formic Acid) and eluted over 90 min with a gradient of 2% B to 30% B in 79 min, ramping to 100% B at 80 min and held at 100% B for the duration of the run. Eluted peptides were sprayed into a ThermoFisher Q-Exactive mass spectrometer using a FlexSpray spray ion source. Survey scans were taken in the Orbi trap (70,000 resolution, determined at *m*/*z* 200) and the top ten ions in each survey scan were then subjected to automatic higher energy collision induced dissociation (HCD) with fragment spectra acquired at 17,500 resolution.

The resulting MS/MS spectra were converted to peak lists using MaxQuant, v1.4.1.2 (www.maxquant.org), and analyzed in the MaxQuant environment [5], [6]. Assignments validated using the MaxQuant maximum 1% false discovery rate (FDR) confidence filter are considered true. Andromeda parameters for all databases were as follows: quantification triple SILAC labeling: light (Arg0, Lys0), medium (Arg4, Lys6), heavy (Arg10, Lys8); allowing maximum 2 missed trypsin sites, fixed modification of carbamidomethyl cysteine, variable modification of oxidation of methionine; peptide tolerance of ±5 ppm, fragment ion tolerance of 0.3 Da and FDR calculated using randomized database search. From each quantified SILAC labeling, three isotope ratios were calculated in each independent experiment.
